# Expression of Glucose Transporter 4 (GLUT4) is Increased by Cinnamaldehyde in C2C12 Mouse Muscle Cells

**DOI:** 10.5812/ircmj.13426

**Published:** 2014-02-04

**Authors:** Abdolrahim Nikzamir, Alireza Palangi, Alireza Kheirollaha, Hashemi Tabar, Alimohamad Malakaskar, Hajieh Shahbazian, Mohammad Fathi

**Affiliations:** 1 Department of Biochemistry, Ahvaz Jundishapur University of Medical Sciences, Ahvaz, IR Iran; 2 Endocrinology and Metabolism Research Center (EMRC), Tehran University of Medical Sciences, Tehran, IR Iran; 3 Cellular and Molecular Research Center, Ahvaz Jundishapur University of Medical Sciences, Ahvaz, IR Iran; 4 Diabetes Research Center, Faculty of Medicine, Ahvaz Jundishapur University of Medical Sciences, Ahvaz, IR Iran; 5 Department of Anesthesiology, Faculty of Medicine, Shahid Beheshti University of Medical Sciences, Tehran, IR Iran

**Keywords:** Glucose Transporter type 4, Cinnamaldehyde, Real-Time Polymerase Chain Reaction, Diabetes Mellitus, Insulin

## Abstract

**Background:**

In diabetes mellitus because of the absence or insufficient sensitivity to insulin, glucose transporter protein in cell membrane, glucose transporter 4, is decreased. GLUT4 is the major glucose transporter in skeletal muscle and adipose tissue, which is under control of insulin. It remains, however, unclear whether cinnamaldehyde plays a regulatory role(s) or not.

**Objectives:**

The objective of this study was to investigate the effects of cinnamaldehyde on GLUT4 gene expression.

**Materials and Methods:**

This study was an experimental trial. Tests were performed in triplicates. This study examined effects of cinnamaldehyde on Glut4 gene expression in C2C12 skeletal muscle cells by using Real Time PCR. C2C12 myoblasts were cultured in DMEM + 10 % FBS. After differentiation of myoblasts to myotubes, the cells were serum deprived for 5 hours and then treated with 10, 20, or 50 µM of cinnamaldehyde for 1 hour.

**Results:**

Our data revealed a significant increase in the expression of Glut4 in cinnamaldehyde treated cells. In addition, GLUT4 mRNA level was increased in a dose dependent manner. Analyses were performed using the SPSS 16 for Windows software. Differences between the groups were determined by one-way ANOVA.

**Conclusions:**

These results demonstrate that cinnamaldehyde up regulates the expression of mouse skeletal muscle GLUT4 gene expression.

## 1. Background

In diabetes mellitus because of the absence of or insufficient sensitivity to insulin, glucose transporter protein in cell membrane called glucose transporter 4 is decreased and this problem leads to a decrease of glucose uptake by cells and therefore results in hyperglycemia (1, 2). Glucose uptake from blood in mammalian cells is accomplished by family of specific membrane transporter proteins called Glut4. Glut4 is one of the proteins whose presence or absence on the cell surface is regulated by insulin. Glut4 exists in many tissues specifically in fat tissue and skeletal muscle. This protein is the main factor required for glucose uptake from plasma and reduction of blood glucose (3, 4). Insulin is involved in regulation of many functions of body organs. One of those functions is glut4 gene expression and locating this protein in fat tissue and skeletal muscle cells that plays a vital role in blood glucose regulation. Glut4 is a glucose transporter protein in tissues specifically in fat and muscle tissue. This protein consists of 509 amino acids and is located on chromosome 17. Glut4 is a membrane-spanning protein which contains 12 trans-membrane domains with intracellular located amino- and carboxyl-terminals (5).

This protein, in its natural form, exists in intracellular vesicles that under insulin stimulation rapidly bind to cell membrane and increase Glut4 protein on cell surface (6). This change causes entrance of glucose to cells and therefore reduces blood glucose. In patients with insulin resistance, this metabolic process is impaired in adipose and muscle tissue and these tissues lose their ability for physiological response to insulin (7). Insulin is a strong attachment inducer of Myocyte Enhancer Factor2 (MEF2) transcription factor to Glut4 gene promoter. Insulin enhances MEF2 binding to DNA by the means of p38 Mitogen Activated Protein Kinase (P38MAPK), Phosphatidyl Inositol3 Kinase (PI3K) and Protein Kinase C (PKC) signaling pathway via IR activation (8, 9). Attachment of MEF2 to DNA is decreased in diabetic patients and related to decline of Glut4 gene expression in these individuals (10).

Cinnamaldehyde (CNA) is a primary constituent found in cinnamon (Cortex cinnamomi). Although antidiabetic and anti-inflammatory activities of cinnamon extract have been investigated in recent years, whether CNA is responsible for these activities is yet to be explored (11). Cinnamaldehyde is one of the major constituents of cinnamomumverum medicinal herb. Significant antihyperglycemic effect of cinnamon has been shown previously (12, 13). Cinnamon contains biologically active substances that have demonstrated insulin-mimetic properties. *In vitro* (14, 15) and *in vivo* (16, 17) studies have shown that cinnamon enhances glucose uptake by activating insulin receptor kinase activity, autophosphorylation of the insulin receptor, and glycogen synthase activity. Other recent studies have demonstrated the ability of cinnamon to reduce lipid levels in fructose-fed rats, potentially via inhibiting hepatic 3-hydroxy- 3-methylglutaryl CoA reductase activity (18, 19). Several clinical trials (6, 20) have investigated the impact of cinnamon on glucose and plasma lipid concentrations in patients with diabetes but yielded conflicting results.

## 2. Objectives

In the present study we investigated the effect of cinnamaldehyde on Glut4 gene expression in C2C12 mouse skeletal muscle cells to elucidate the molecular basis of antidiabetic potential of cinnamaldehyde.

## 3. Materials and Methods

This study was an experimental trial. C2C12 cells (*Mus musculus* c3h) were obtained from the Iranian branch of the Pasteure institute. Dimethylesulfoxide (DMSO), sodium dudecil sulfate (SDS), guanidine hydrochloride, Trypsin-Ethylene Diamine Tetra Acetic acid (EDTA) solution and penicillin/streptomycin solution were purchased from Sigma-Aldrich, USA. Cinnamaldehyde, ethanol, NaCl, KCl, Na2HPO4 and KH2PO4 were obtained from Merc, Germany. Nuclease free water, gDNA wipe out buffer, fetal bovine serum (FBS), sodium pyruvate and Dulbecco’s Minimum Essential Medium (DMEM) were purchased from Gibco, USA. Tripure isolation reagent, cell death detection kit and cytotoxicity detection kit were obtained from Roche, Germany. Quntifast SYBR green real time PCR kit and quntitect reverse transcription kit were obtained from Qiagen, USA. Di Ethyl Pyro Carbonate (DEPC) water was purchased from Cinnagen, Iran. Primer sequences were synthesized by Cinnagen, Iran.

### 3.1. Cell Culture

C2C12 cells were cultured primarily in DMEM medium supplemented with 10% FBS, 2 mM Glutamine, 110 mg/L sodium pyruvate, and sodium bicarbonate (3.7 g/L) at 37°C and 5% CO_2_. Penicillin (100 U/ml), streptomycin (100 mg/ml) and Amphotericin B (2.5 mg/L) were added to the culture media for inhibition of bacterial and fungal contamination. Every two days and at the confluent stage, cells were trypsinized and subcultured in three new flasks at a density of 1 × 10^6^ cells per 25 cm^2^ flask (21).

### 3.2. Cell Differentiation

For differentiating the cells to myotube, cells were grown to confluence and medium was changed to DMEM supplemented with 4 mM Glutamine, 110 mg/L sodium pyruvate, 3.7 g/L sodium bicarbonate, 100 U/ml penicillin, 100 mg/ml streptomycin, 2.5 mg/L amphotericin B and 2% horse serum. Cells were incubated with this medium for two weeks and every two days culture medium was substituted with fresh culture medium until cells fully differentiated to myotubes (21).

### 3.3. Cinnamaldehyde Cytotoxicity Detection

The viability of cells that were treated with 10, 20, 50, 100 µM of cinnamaldehyde in culture medium were quantified by investigation of LDH release from these cells as described bellow (22).

C2C12 cells were cultured in a 96 well cell culture plate. After 48 hours, incubation of cells in serum free medium, different concentrations of cinnamaldehyde were added to each well from the stock solution of 0.5 M cinnamaldehyde in DMSO (the medium final concentration was less than 0.1%) (23) for 1 hour. Then the viability of cells was quantified by measuring the activity of released LDH from cells with a spectrophotometric assay by the Biorad model 680 microplate reader. All experiments were accomplished in triplex test.

### 3.4. Cinnamaldehyde Treatment

Cells were divided in to four groups. The control group was treated by DMSO (final concentration was less than 0.1 %). The other three groups were treated with 10, 20, or 50 µM of cinnamaldehyde for 1 hour.

### 3.5. RNA Extraction

Total RNA from different experimental conditions was extracted from C2C12 cells using the tripure isolation kit (Roche diagnostic, Germany) (24). The concentration and purity of the obtained RNA was determined by quantitation of 260/230 nm absorbance ratio and 280/260 absorbance ratio (25).

### 3.6. cDNA Synthesis

Extracted RNA was treated by gDNA wipe out buffer (Qiagen, USA), to completely remove possible existing genomic DNA, and then 1 µg of pure RNA was reverse transcribed to cDNA using the quantiscript cDNA synthesis kit (Qiagen, USA). Reverse transcription was performed at 42°C for 15 minutes followed by incubation for 3 minutes at 95°C to inactive the RT enzyme. cDNA was stored at -70°C until it was used.

### 3.7. Relative Quantitation by Real Time PCR

Nucleotide primers for scl2a4 (Glut4) and Glycer Aldehydes 3 Phosphate Dehydrogenase (GAPDH) were designed by the primer 3 software and blasted in the NCBI Pubmed primer blast software. Forward and reverse primer sequences were:

Forward primer: 5´CAAAGCATCGACCAGTGCTA3´ and reverse primer: 5´TGGACAGCACTGACTTCCAG3´ for GAPDH, and forward primer: 5´GAGCCTGAATGCTAATGGAG3´ and reverse: 5´GAGAGAGAGCGTCCAATGTC3´, for Glut 4.

### 3.8. Real Time PCR

Glut4 gene expression was measured by a comparative CT method (ΔΔCT) real time PCR using the ABI step one plus instrument with Quantifast SYBR green kit (Qiagen, USA). GAPHD expression was used as an internal control. Before using the ΔΔCT method for quantitation, a validation experiment was performed for primer efficiency investigation. Real time PCR was performed under the following conditions; 40 cycles during 5 minutes at 95°C for hotstar taq polymerase activation, 10 seconds at 95°C for denaturation and 30 seconds at 60°C for annealing and extension.

### 3.9. Statistical Analysis

All results are presented as means ± SD Differences between the groups were determined by one-way ANOVA, with Post-Hoc comparison by Tukey multiple comparison test. The level of significance for all statistical analyses was set at P < 0.05. Analyses were performed using SPSS 16 for Windows software.

## 4. Results

### 4.1. Cinnamaldehy Cytotoxicity

**Figure 1.**
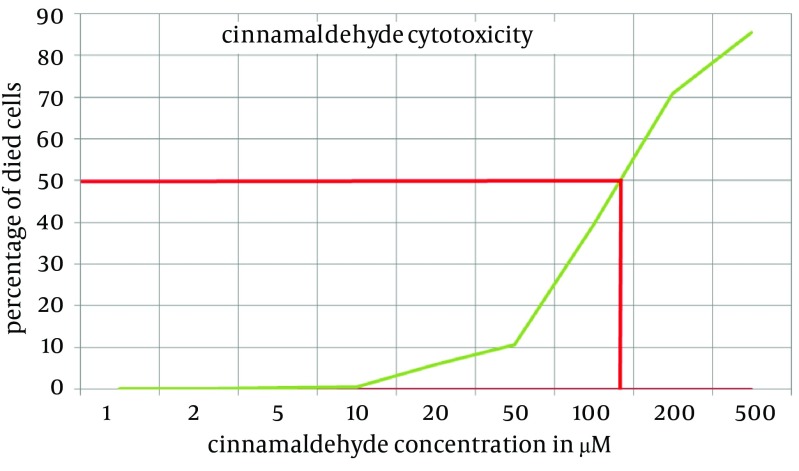
Cynnamaldehyde Toxic Dose Determination

To examine the cinnamaldehyde cytotoxicity or to check for cell viability, the LDL assay was performed by incubation of C2C12 cells with different concentration of cinnamaldehyde for 1 hour. The data presented in Figure 1 reveals that cytotoxicity of cinnamaldehyde is very low at 50 µM; about 10%, and 5 % of cells died at 140 µM of cinnamaldehyde.

### 4.2. Primer Efficiency and mRNA Expression

**Figure 2.**
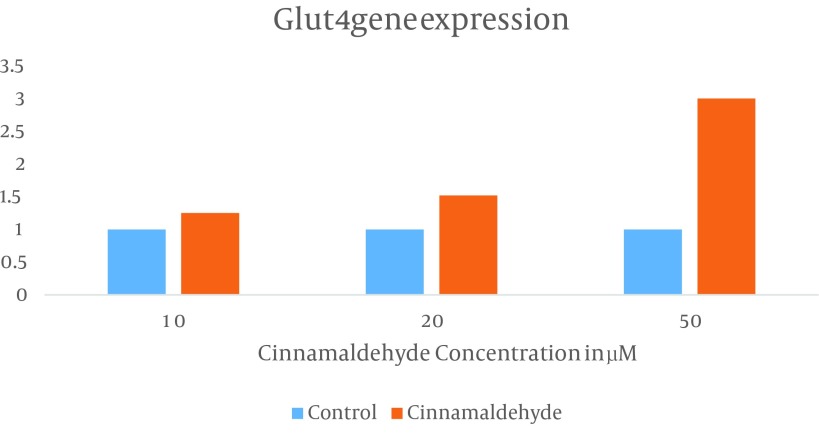
The effect of Cynnamaldehyde on Glut4 mRNA

First primer efficiency was checked and the slope of the log input of cDNA amount VS ΔCT was less than 0.1, which is an acceptable outcome. Next, to determine the *in vitro* effect of cinnamaldehyde on gene expression of Glut4, we preformed a RT-PCR experiment by using Glut4-specific primers. Quantitative RT-PCR analysis revealed that Glut4 mRNA was significantly increased by cinnamaldehyde treatment in a dose dependent manner compared to the cells treated with vehichl, DMSO. Major increase (three-fold) in the level of Glut4 mRNA was observed when the cells were treated with 50 µM of cinnamaldehyde for one hour (Figure 2). Data were statistically analyzed by one-way ANOVA followed by Tukey’s multicomparison test and summarized in Table 1. There was a significant difference between the mRNA levels of Glut4 in the cells treated with cinnamaldehyde compared to the controls (P ≤ 0.05).

**Table 1.** Real Time PCR Results ^a, b^

Sample	CT gene GluT 4	CT gene GAPDH	ΔCT (GluT 4-GAPDH)	ΔΔCT Control (ΔCT - ΔCT)	2- ΔΔCT
**Control cells**	32.680 ± 0.09	21.243 ± 0.04	11.437 ± 0.1	0.0 ± 0.1	1.0 (0.9-1.1)
**Treated cells with cinnamaldehyde 10 µM**	29.896 ± 0.04	18.788 ± 0.05	11.158 ± 0.06	− 0.329 ± 0.06	1.256 (1.250-1.261)
**Treated cells with cinnamaldehyde 20 µM**	28.878 ± 0.02	17.130 ± 0.07	10.748 ± 0.07	− 0.682 ± 0.07	1.604 (1.596-1.612)
**Treated cells with cinnamaldehyde 50 µM**	25.342 ± 0.04	15.496 ± 0.03	9.846 ± 0.05	− 1.591 ± 0.05	3.012 (3.002-3.023)

^a^Data are means ± SD

^b^ The effects of cinnamaldehyde (10 µM, 20 µM, and 50 µM) on mRNA levels of GluT 4 in c2c12 muscle cells. mRNA levels were analyzed by the cyber green procedure. Results represent means± SD from triplicate determinations, representative of 3 independent experiments; four to six experiments compared with control. Statistically significant differences between treatments are indicated by one-way ANOVA followed by Tukey’s multicomparison test. In comparison with controls, all treatments were significant (P ≤ 0.05)

## 5. Discussion

The relationship between the Cinnamon and type 2 diabetes mellitus has been the subject of many recent studies. It has been proved that drug plants are full of active components that have medical effects (26). Plenty of components derived from plants are proved to cure type 2 diabetes mellitus (27). One of those plants is cinnamon, and consists of various constituents. Cinnamon is a sweet, aromatic, aphrodisiac plant that is useful in treatment of bronchitis, asthma, cardiac disease, diarrhea, uropathia and fever (28). Important ingredients of cinnamon are cinnamaldehyde, cinnamic acid, cinnamyl alcohol, polyphenoles and hydroxyl chalcone. Many ingredients of cinnamon have been studied until its main constituent with antidiabetic effects and insulin mimetic ability was identified. Cinnamic acid, cinnamon drived hydroxyl chalcone, water-soluble polyphenoles of cinnamon and cinnamaldehyde in different studies, have shown antidiabetic effects (29).

Cinnamaldehyde is a major substance in cinnamon and it has been proved that this substance is useful for blood glucose and fat reduction in diabetes induced by streptozotocin in rats (29, 30). Skeletal muscles have an essential role in body energy balance and they are the premier tissues that are affected by insulin for glucose uptake. Therefor, they are appropriate target tissues for non-insulinic diabetes mellitus drugs (30) and so in this study mouse skeletal muscle cell line, C2C12, was used. Since the enhancement of Glut4 (31) has the most effect on insulin in fat and muscle tissues, for reduction of blood glucose, in this study the effect of cinnamaldehyde on gene expression of Glut4 in C2C12 was investigated. Shaohui et al. reported that cinnamaldehyde is a strong reducer of blood glucose in diabetic Wistar rats. They also indicated that this compound increases plasma insulin level. Zhang et al. showed that the protein level of Glut4 was increased in fat, liver, and muscle tissues of diabetic rats by long term treatment with high dose of cinnamaldehyde (32). Our findings showed that Glut4 expression is increased after a short duration treatment with cinnamaldehyde in a dose dependent manner in C2C12 cells. Our research results determined that Glut4 mRNA in C2C12 cells treated with 10, 20, and 50 µm of cinnamaldehyde increased by 1.25, 1.6, and 3.01 folds, respectively compared to control cells that were treated with DMSO. These results show that cinnamaldehyde increases Glut4 gene expression with short-term treatment in C2C12 cell line.

These findings are in line with the research results of Zhang et al. They showed that high dose and oral consumption of 40 mg cinnamaldehyde per kilogram body weight, for four weeks, in diabetic rats, increased Glut4 protein by 2 folds (32). However, they did not show whether this increase in the level of Glut4 protein is due to the increase in its mRNA or reduction of its degradation. The present data led us to conclude that up regulation of Glut4 may cause an increase of its protein level, previously reported by others. These results show that oral consumption of cinnamaldehyde may cause reduction in the effectiveness of Glut4, which is probably because of insufficient absorption. In the present research, we investigated the effect of cinnamaldehyde on Glut4 mRNA level, yet Zhangwei measured Glut4 protein level and since the entire transcribed mRNA does not translate to protein, the same results were not achieved. Our results showed a three-fold increase in Glut4 mRNA level and Zhang results showed a two-fold increase in Glut4 protein level.

Also we studied the short-term effect and zhang studied the long term effect of cinnamaldehyde on Glut4. Another reason leading to the different results is that the sample that was utilized in our research was *in vitro *and in the Zhang research this was *in vivo*. Although there are increasing reports in agreement with our data (31, 32), Blevins et al. showed that cinnamon has no effect on blood glucose (33). Reasons that explain why blood glucose was not affected by cinnamon in Blevins research could be related to sample society, dose, and type of cinnamon consumption. Blevins et al. noted that cinnamon has various effects on different human races that could lead to the different results. On the other hand, daily oral consumption of 1 gr of cinnamon was perhaps not a sufficient dose for reducing the blood glucose. In conclusion, we reported that cinnamaldehyde causes enhancement in Glut4 expression in C2C12 muscle cells, and this can be accounted for the blood glucose reduction through glucose utilization by muscle tissue. So, the effect (s) of cinnamaldehyde on the up regulation of Glut4 may be useful for treating patients with insulin insufficiency or insulin resistance, however the nature and mechanism of these effects needs to be investigated.
